# The Use of Smart Technology in an Online Community of Patients With Degenerative Cervical Myelopathy

**DOI:** 10.2196/11364

**Published:** 2019-05-09

**Authors:** Oliver Daniel Mowforth, Benjamin Marshall Davies, Mark Reinhard Kotter

**Affiliations:** 1 Division of Neurosurgery Department of Clinical Neurosciences Addenbrooke's Hospital, University of Cambridge Cambridge United Kingdom

**Keywords:** spinal cord diseases, cervical vertebrae, spinal osteophytosis, spondylosis, biomedical technology, chronic disease, follow-up studies

## Abstract

**Background:**

Degenerative cervical myelopathy (DCM) is a prevalent and progressively disabling neurological condition. Treatment is currently limited to surgery, the timing of which is not without controversy. New international guidelines recommend that all patients should undergo lifelong surveillance and those with moderate-to-severe or progressive disease should be offered surgery. Long-term surveillance will place substantial burden on health services and short clinic assessments may risk misrepresenting disease severity. The use of smart technology to monitor disease progression could provide an invaluable opportunity to lessen this burden and improve patient care. However, given the older demographic of DCM, the feasibility of smart technology use is unclear.

**Objective:**

The aim of this study was to investigate current usage of smart technology in patients with self-reported DCM to inform design of smart technology apps targeted at monitoring DCM disease progression.

**Methods:**

Google Analytics from the patient section of Myelopathy.org, an international DCM charity with a large online patient community, was analyzed over a 1-year period. A total of 15,761 sessions were analyzed.

**Results:**

In total, 39.6% (295/744) of visitors accessed the website using a desktop computer, 35.1% (261/744) using mobile, and 25.3% (188/744) using a tablet. Of the mobile and tablet visitors, 98.2% (441/449) utilized a touchscreen device. A total of 51.3% (141/275) of mobile and tablet visitors used iPhone Operating System (iOS) and 45.8% (126/275) used an Android operating system. Apple and Samsung were the most popular smart devices, utilized by 53.6% (241/449) and 25.8% (116/449) of visitors, respectively. The overall visitor age was representative of DCM trials. Smart technology was widely used by older visitors: 58.8% (113/192) of mobile visitors and 84.2% (96/114) of tablet visitors were aged 45 years or older.

**Conclusions:**

Smart technology is commonly used by DCM patients. DCM apps need to be iOS and Android compatible to be accessible to all patients.

## Introduction

### Background

Degenerative cervical myelopathy (DCM) is a chronic and progressive neurological condition of symptomatic spinal cord compression secondary to degenerative changes in the cervical spine [1,2].

In classical descriptions, DCM patients present complaining of a broad-based gait and clumsy hands [3-5]. In reality, symptoms are varied and often subtle, which contributes to significant underdiagnosis, misdiagnosis, and delayed diagnosis [6]. This has hindered accurate characterization of its epidemiology, but based on imaging studies, the prevalence of DCM could be as high as 5% in those over 40 years old [6]. Ultimately, the condition is progressive and in extreme circumstances can lead to paralysis [5]. This significant disability severely impacts quality of life; a recent study found that quality of life in DCM patients is lower than in almost any other chronic disease, including cancer, diabetes, and chronic lung disease [7].

At present, surgical decompression is the only effective treatment for DCM. It is able to halt disease progression and provide some degree of improvement. However, despite surgery, most patients will continue to suffer from neurological deficits [8,9], and therefore, the timing of surgery is crucial [10]. If offered too late, this will expose patients to irreversible damage; if offered prematurely, surgery may expose patients to an invasive procedure with a risk of potential unintended effects, such as adjacent segment degeneration leading to future DCM development at nonoperated spinal levels.

Consequently, there is an increasing need for close monitoring of patients with DCM. New international guidelines recommend that for moderate-to-severe disease, surgery should be offered and patients should be monitored after surgery [10]. For mild disease, long-term follow up is recommended [10]. Surveillance of this large and increasing cohort of patients poses many problems, including a huge burden on health services. Moreover, snapshot outpatient clinic assessments once or twice per year risk misrepresenting disease severity. In addition, current disease severity measures are poorly sensitive to change and poorly adapted to research studies, limiting outcomes for both present and future patients [11].

Technological advances, especially smart technologies such as mobile phones, offer a novel and innovative solution to this problem. Smart technology is increasingly prevalent in the general population: in 2017, it was estimated that there were almost 4 billion internet users worldwide [12]. Moreover, a recent survey found that 85% of the adult population of the United States own a mobile phone and 45% own a smartphone [13]. A study of 300 participants seeking health care in a US emergency department found that 71% owned smartphones, of which 95% had apps and 44% had health apps [14].

Smart devices have highly sophisticated inbuilt technologies, including global positioning systems, accelerometers, microphones, speakers, and cameras capable of fulfilling medical assessments [15]. Smart technology has average to excellent accuracy in measuring a range of physical activities including differentiation of static activity, stair climbing, cycling, walking, and running [16], allowing widespread use of smartphone apps in measuring biological parameters, such as in diabetes, in cardiac rehabilitation, and falls in the elderly [15].

Smart technologies allow users to input data at high frequency, making it much easier to detect change with time, which is important in DCM. Current DCM disease severity measures are relatively simple, focusing largely on gait and motor functioning, making them highly accessible for patients to understand and accurately score [17,18] and highly compatible with a mobile smart device. Moreover, users appear motivated to engage with smart technology: 52% of smartphone users reported using their smartphone to search for health information [13].

Such assessment tools may have additional benefits, transferring assessments to nonspecialists to facilitate earlier diagnosis and may offer a useful research tool. There are also clear financial benefits; a report for the European Union estimated that mobile health (mHealth) could save 99 billion euros in health care costs in the European Union and add 93 billion euros to the European Union’s gross domestic product in 2017 if its adoption is encouraged [19].

Owing to its degenerative nature, the average age of patients undergoing surgery for DCM is in the mid-50s [2]. Currently available clinical trial data suggest that the DCM patient demographic is approximately 60% male and 80% Caucasian with the mean age of presentation reported as 56 to 64 years [20,8]. Clearly, for smart technology to offer an immediate benefit, it would need to be accessible to a high proportion of patients. Whether this is feasible for the current DCM population is unknown.

### Objectives

This study aimed to assess the current usage of smart technology in patients with DCM to ascertain the feasibility of introducing a smart technology–based assessment tool. Specifically, we aimed to establish the relative use of smart technologies (mobile phones and tablets) and traditional desktop devices by patients with DCM to engage with Myelopathy.org, a health charity specifically designed for DCM patients. We hypothesized that smart technology is utilized by DCM patients of all age groups.

## Methods

### Study Design

A cross-sectional observational study was conducted. All reporting adheres to the Strengthening the Reporting of Observational Studies in Epidemiology (STROBE) guidelines from the Enhancing the Quality and Transparency of Health Research (EQUATOR) Network [21].

### Setting

**Figure 1:**
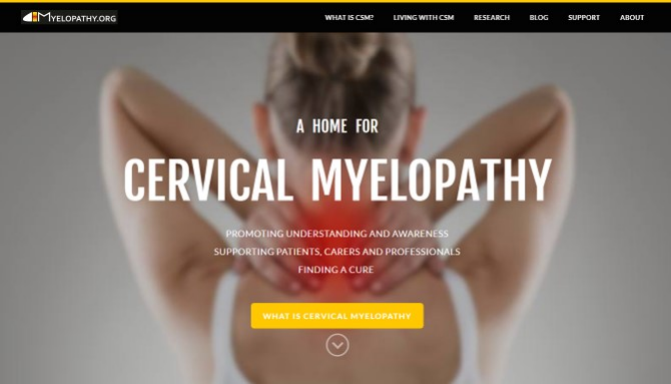
Homepage screenshot from Myelopathy.org, an international myelopathy charity.

Data on visitor demographics to the patient section of Myelopathy.org (Figure 1), an international myelopathy charity, were collected over a 1-year period from April 2016 to April 2017 using Google Analytics (Google). Myelopathy.org is designed for patients, professionals, and carers and has a growing online community.

**Figure 2:**
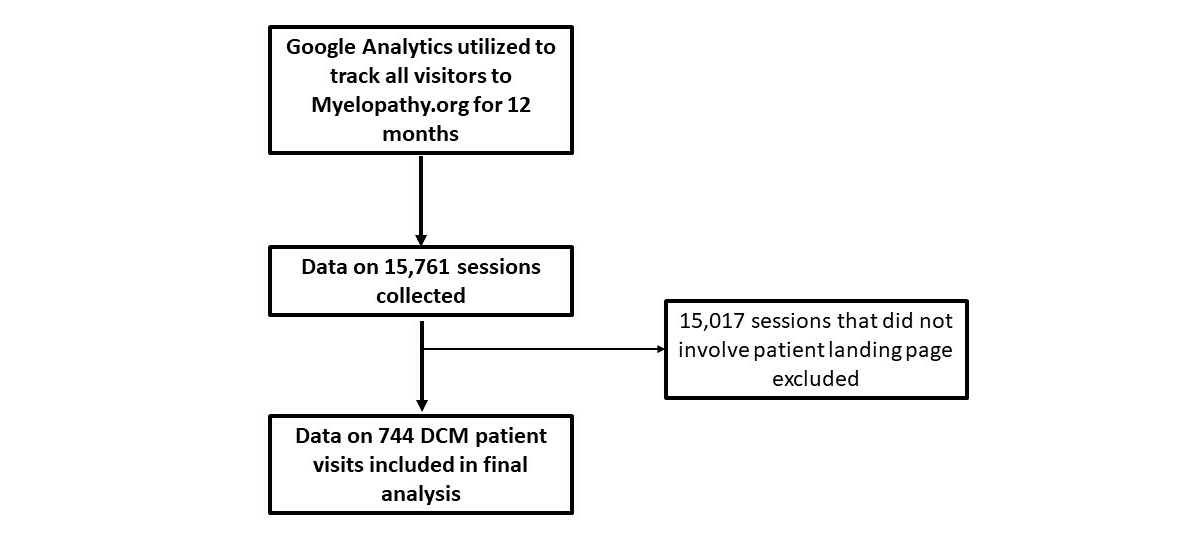
A total of 15,761 sessions were analysed, 4.7% (744) of which involved the degenerative cervical myelopathy patient survey page. DCM: degenerative cervical myelopathy.

### Participants

Patients with DCM were identified by selecting visitors who accessed an e-survey landing page, intended for patients, hosted by Myelopathy.org [22]. This unique landing page required visitors to click through a description of the disease to confirm they had a diagnosis of DCM. A total of 15,761 website visiting sessions were analyzed. Sessions were undertaken by 10,294 visitors, of which 10,261 were new visitors. Although many of the discarded visits were likely from patients, only the 744 visits that involved clicking through to the patient landing page from the main website were included in the analysis to ensure greater certainty that included visits were from patients (Figure 2).

### Data Sources

All data were extracted directly from Google Analytics. Data were analyzed using Microsoft Excel (Office 365, Microsoft).

### Variables

Variables of interest extracted from Google Analytics were sessions (visits); users; demographics including age, gender, and location; device use and device characteristics including mobile and tablet operating systems; mobile and tablet manufacturer; and mobile and tablet input selector.

### Bias

No visitors were excluded. As a self-selected population, it is possible that nonpatient visitors were included in the total website visits. We suspected that most website visitors were patients: for example, approximately 800 complete survey responses were received from patients and 50 from carers to similar surveys over the same time period. Nonetheless, using only visits to the patient landing page increases the certainty of including only patients in our analysis. Given this study design, we believed any nonpatient influence was likely to be very small and, in the context of our large sample size, was unlikely to influence the overall results. To mitigate against ascertainment selection bias, we included a subsection with total website visitor data in the Results section; the similarity of these data to the patient landing page data provides some reassurance against selection bias and suggests that most website visitors are likely to be patients.

### Study Size

Overall 43,004 page views from 15,761 visits were analyzed. A total of 10,261 new visitors were analyzed, of which 744 visited the patient landing page via the Myelopathy.org homepage. All DCM patient visitors over the 12-month study period were included.

### Statistical Methods

Formal statistical analysis was deemed inappropriate.

### Ethics Approval and Consent

The study was ethically approved by the Cambridge Human Biology Research Ethics Committee, University of Cambridge.

## Results

### Participant Demographics

Key demographic characteristics of DCM patient visitors are summarized in Table 1. In total, 29.8% (145/487) of visitors were male. The age range was broad from 18 years to over 65 years. The overall visitor location was diverse. Patient visitors came from over 31 different countries, predominantly the United States (34.1% (254/744)) and the United Kingdom (53.8% (400/744)), representing 87.9% (654/744) of overall visitors.

### Smart Technology Use

#### Device

The Myelopathy.org patient survey was accessed by desktop, mobile, and tablet devices. A total of 35.1% (261/744) of visitors accessed the survey using a mobile phone, 39.6% (295/744) using a desktop device, and 25.3% (188/744) using a tablet device.

#### Smart Technology Users

Of the smart technology (mobile and tablet) visitors, 98.2% (441/449) utilized a touchscreen device. Although iPhone Operating System iOS (51.3% (141/275) and Android (45.8% (126/275) operating systems were dominant in their share of visitors, with a combined 97.1% (267/275) of patient visitors utilizing 1 of the 2 operating systems, use by device manufacturer was more diverse. Although Apple (53.6% (241/449)) and Samsung (25.8% (116/449)) were the most popular device manufacturers, 20.6% (92/449) of devices were produced by 22 other manufacturers. No manufacturer other than Apple or Samsung was utilized by more than 2.5% (11/449) of visitors, and 86.4% (388/449) of visitors utilized devices from one of the top 5 most popular manufacturers, including LG (2.5% (11/449)), Amazon (2.5% (11/449)), and Motorola (2.0% (9/449)), in addition to Apple and Samsung.

### Smart Technology Engagement Across Age Groups

Overall, the visitor age range was broad (Table 1), with 68.0% (328/482) of visitors aged 45 years or older. The overall modal visitor age group was 45 to 54 years.

**Figure 3:**
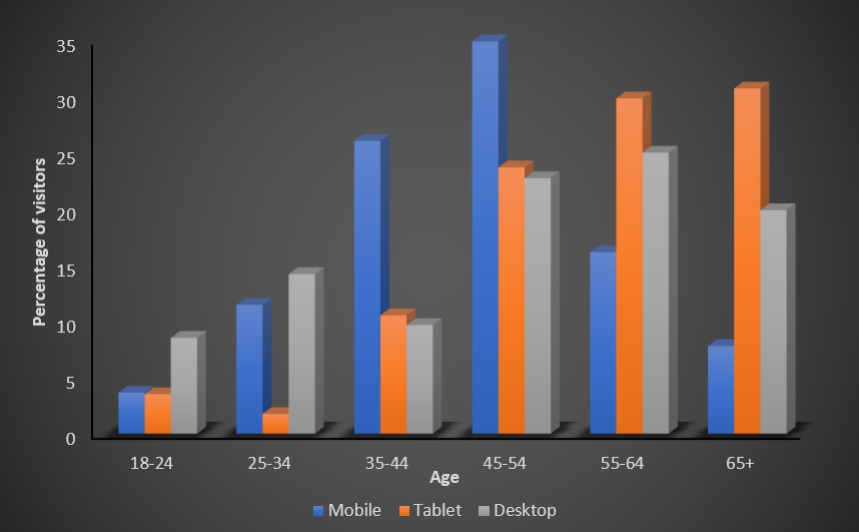
Percentage age distributions of total visits using each device. The percentage of visitors in each age group differed depending on device used.

The patient visitor profile for each technology according to age is shown in Figure 3. The modal age group was 45 to 54 years for mobile visitors, 65+ years for tablet visitors, and 55 to 64 for desktop visitors. All 3 device types were widely used among older patients, with 58.8% (113/192) of mobile, 84.2% of tablet (96/114), and 67.6% (119/176) of desktop visitors aged 45 years or older. Of all tablet visitors, the number of visitors per age group increased with age, up to a peak in the modal 65+ age group. The number of desktop visitors per age group also tended to increase with age, whereas for mobile devices, the number of visitors per age group increased with age up to the modal age group of 45 to 54 years, before declining in older age groups.

Gender composition was 29.8% (145/487) male (Table 1). The modal age of both male and female patient visitors was 45 to 54 years. The number of visitors increased as age increased for both sexes, with males plateauing from the 35 to 44 years age group whereas female visitors showing a clear peak at the 45 to 54 years group.

Almost identical age distributions were seen between visitor populations from the United States and the United Kingdom. The modal age group was 55 to 64 years for US patient visitors and 45 to 54 years for UK patient visitors. A total of 75.0% (132/176) of US patient visitors were aged 45 years or older, whereas 64.0% (162/253) of UK visitors were aged 45 years or older.

### Comparison of Visitors to the Patient Landing Page and All Website Visitors

Visitors to the patient landing page were similar to overall website visitors for variables of interest (Table 2). In particular, age and gender demographics were similar, as were data on device and mobile operating system and manufacturer. Although percentages of visitors from each of the 5 most common locations showed some variation, for both groups the United Kingdom and the United States were the most prevalent locations, followed by Canada, Australia, and India.

**Table 1:** Demographic characteristics of visitors to the patient survey page of Myelopathy.org.

Demographic characteristic		Total, n (%)	Mobile, n (%)	Tablet, n (%)	Desktop, n (%)
**Age (years)**					
	18-24	26 (5.4)	7 (3.7)	4 (3.5)	15 (8.5)
	25-34	49 (10.2)	22 (11.5)	2 (1.8)	25 (14.2)
	35-44	79 (16.4)	50 (26.0)	12 (10.5)	17 (9.7)
	45-54	134 (27.8)	67 (34.9)	27 (23.7)	40 (22.7)
	55-65	109 (22.6)	31 (16.1)	34 (29.8)	44 (25.0)
	65+	85 (17.6)	15 (7.8)	35 (30.7)	35 (19.9)
**Gender**					
	Male	145 (29.8)	53 (27.6)	22 (19.1)	70 (38.9)
**Visitor location**					
	United States	254 (34.1)	107 (41.0)	58 (30.9)	89 (30.2)
	United Kingdom	400 (53.8)	117 (44.8)	120 (63.8)	163 (55.2)
	Canada	29 (3.9)	10 (3.8)	4 (2.1)	15 (5.1)
	Australia	12 (1.6)	5 (1.9)	3 (1.6)	4 (1.4)
	India	6 (0.8)	4 (1.5)	0 (0)	2 (0.7)
	Ireland	6 (0.8)	2 (0.8)	3 (1.6)	1 (0.3)
	Malaysia	4 (0.5)	3 (1.2)	0 (0)	1 (0.3)
	Other	33 (4.5)	13 (5.0)	0 (0)	20 (6.8)

**Table 2:** Comparison of visits to patient landing page and all website visits.

Variables		Patient landing page, n (%)	Website, n (%)
**Visitors**			
	Sessions (visits)	744	15,761
	New visitors	478 (64.3)	10,261 (65.1)
	Returning visitors	266 (35.7)	5500 (34.9)
**Age (years)**			
	18-24	26 (5.4)	621 (6.4)
	25-34	49 (10.2)	1504 (15.5)
	35-44	79 (16.4)	1811 (18.7)
	45-54	134 (27.8)	2527 (26.1)
	55-64	109 (22.6)	2088 (21.5)
	65+	85 (17.6)	1145 (11.8)
**Gender**			
	Female	342 (70.2)	6483 (66.1)
	Male	145 (29.8)	3331 (33.9)
**Visitor location**			
	United States	254 (34.1)	6491 (41.2)
	United Kingdom	400 (53.8)	5768 (36.6)
	Canada	29 (3.9)	813 (5.2)
	Australia	12 (1.6)	382 (2.4)
	India	6 (0.8)	280 (1.8)
	Other	43 (5.8)	2027 (12.8)
**Device**			
	Desktop	295 (39.6)	6795 (43.1)
	Mobile	261 (35.1)	6311 (40.0)
	Tablet	188 (25.3)	2655 (16.9)
**Mobile operating system**			
	iPhone Operating System	141 (51.3)	3593 (57.3)
	Android	126 (45.8)	2531 (40.4)
	Other	8 (2.9)	147 (2.3)
**Mobile device manufacturer**			
	Apple	241 (53.6)	5161 (57.5)
	Samsung	116 (25.8)	1915 (21.4)
	LG	11 (2.5)	221 (2.5)
	Motorola	9 (2.0)	197 (2.2)
	Amazon	11 (2.5)	144 (1.6)
	Other	61 (13.6)	1328 (14.8)
**Mobile input selector**			
	Touchscreen	441 (98.2)	8640 (96.4)
**Desktop age stratification (years)**			
	18-24	15 (8.5)	399 (11.1)
	25-34	25 (14.2)	758 (21.0)
	35-44	17 (9.7)	514 (14.3)
	45-54	40 (22.7)	705 (19.6)
	55-64	44 (25.0)	684 (19.0)
	65+	35 (19.9)	540 (15.0)
**Mobile age stratification (years)**			
	18-24	7 (3.7)	185 (4.2)
	25-34	22 (11.5)	662 (15.0)
	35-44	50 (26.0)	1110 (25.1)
	45-54	67 (34.9)	1431 (32.4)
	55-64	31 (16.1)	794 (18.0)
	65+	15 (7.8)	235 (5.3)
**Tablet age stratification (years)**			
	18-24	4 (3.5)	37 (2.2)
	25-34	2 (1.8)	84 (5.0)
	35-44	12 (10.5)	187 (11.1)
	45-54	27 (23.7)	391 (23.3)
	55-64	34 (29.8)	610 (36.3)
	65+	35 (30.7)	370 (22.1)

## Discussion

### Principal Findings

The use of smart technology is prevalent in patients of all ages with DCM, with patients favoring portable devices such as mobiles and tablets. The distribution of technology usage across age groups differed, with mobiles favored in middle age and tablet and desktop usage more common in later years. Android and iOS are the predominant mobile operating systems utilized by patients with DCM.

### Generalization of Findings

From the outset, it is important to consider the limitations of this study and, in particular, whether this population represents DCM as a whole; an internet platform is a self-selected population, both in terms of confirming the diagnosis of DCM and for which access requires technology usage.

Although this is a potential limitation, it is important to recognize that internet usage among older age groups is well described [23] and the focus was instead the use of smart technology, for which desktop visitors could act as a surrogate control group.

In addition, visitor age was representative of DCM trials, which frequently report a mean patient age of 56 years [8,9]. In this study, 40.2% (194/482) of overall visitors were aged 55 years or older and the modal visitor age was 45 to 54 years. Unfortunately, owing to the limitations of Google Analytics, age is presented in age ranges and the age group 65+ years is particularly broad and would benefit from subanalysis.

Although the gender constitution differed (29.8% (145/487) male compared with trial populations of between 60% and 65% male [8,9]), gender did not influence technology usage in this study and thus is unlikely to have substantially influenced results. In addition, it has been shown that women utilize the internet for health information and support more widely than men [24,25]. We have previously shown that this relates to the weighting of Facebook patient support groups, which are predominantly female and which were the most successful recruitment strategy [22]. Therefore, increasing male participation in DCM electronic health (eHealth) initiatives likely requires an approach much broader than anything targeted specifically at DCM. Nonetheless, targeted advertising, information leaflets, and signposting in hospital outpatient clinics may form first steps. Moreover, current development of a DCM repository to directly capture data from health records will help validate internet survey data. Finally, a designated patient-run section of Myelopathy.org, Myelopathy Support, has a large Web-based patient following and appears to be encouraging males to engage.

There is a small risk that some visitors included did not in fact have DCM. However, the focus on those visitors accessing a patient survey, in which they had to click through a description of symptoms to confirm they had a diagnosis of DCM before accessing the patient survey page alongside the rational demographics, makes this unlikely. In addition, we have previously shown that, at present, the platform is infrequently used by nonpatients [26]. Therefore, any contribution from non-DCM patients is likely to be small and have a negligible influence on results. The similarity of data on visitors to the patient landing page and data on all website visitors provides reassurance that any selection biases likely had a negligible influence on results and suggests that the total website visitors were mostly patients.

In addition, although visitors were mostly from the United States or the United Kingdom, visitors were also from across the globe, showing potential for smart technology elsewhere.

### The Emerging Role of Smart Technology for Health, Including Degenerative Cervical Myelopathy

The potential of smart technology for health is rapidly becoming established. There has been an explosion of health-related smart technology apps, aimed at a multitude of areas including education, medical assessment, and medical intervention [15].

To date, much focus has been on educational apps for both patients and professionals. In addition, apps have incorporated simple assessments, the inputting of survey data, simple sensor metrics, such as physical activity, and partnership with third-party technologies, such as blood sugar monitors [27]. However simple, their usage is being shown to have a significant clinical benefit with easy uptake by patients. For example, a recent systematic review of mobile and smart technology use in diabetes care found that the majority of interventions improved primary endpoints such as HbA1c and that technologies that interacted with both patients and providers were most likely to be successful [28]. A public opinion survey applied to a Greek population found strong significant effects of perceived usefulness, relative advantage of use, and perceived ease of use of a smart mHealth app [29]. Moreover, Anderson et al utilized a semistructured interview format to explore how health consumers use apps for health monitoring in Australia. They found that apps were used to monitor conditions including diabetes, asthma, depression, coeliac disease, blood pressure, migraine, and menstrual cycle irregularity on an approximately weekly basis [30]. This clear potential and appetite is contributing to significant investment and rapid growth in mHealth [19], including more sophisticated systems.

This includes patients with DCM, where new guidelines [10] advise close surveillance; however, current clinical assessments have shown poor responsiveness to change [11]. Laboratory gait analysis is a quantitative assessment, which is showing promise to overcome these limitations as it can provide a sensitive and reproducible measure of walking [31], is able to detect subtle progression [32], can distinguish patients from healthy controls [33], can predict outcome after surgery [34], and can predict disease progression [35,36]. Researchers in the field of Parkinson’s disease have demonstrated that a similar analysis can occur using smartphones [37-39], which would offer the additional benefit of continuous monitoring in a patient’s own environment.

Clearly, a significant barrier to the uptake of any potential smart technology app would be its accessibility to the target audience. Therefore, the prevalence of smart technology among patients with DCM in this study is a reassuring finding. However, it is worth noting that not all smart technology has the same eHealth potential; for example, although desktops have input capabilities, they may lack monitoring sensors; tablets may have similar monitoring capabilities as smartphones, but their larger size may preclude certain measurements such as pocket-mediated gait analysis. In addition, owing to the requirement for Web browsing, this study has not captured the use of smart watches, which also have significant mHealth potential [40].

### Conclusions

Smart technology use is prevalent in patients with DCM. An app to monitor DCM disease severity must be compatible with both iOS and Android and multiple device manufacturers. For greatest immediate uptake, both phone and tablet compatibility are desirable, although this must be considered in the context of an app’s objectives. Although such an app is yet to be developed, this study has shown that the user group is at least in possession of the necessary technology and has the willingness to use it.

## Abbreviations

DCM: degenerative cervical myelopathy
eHealth: electronic health
iOS: iPhone Operating System
mHealth: mobile health
